# Cancer registration in the Middle East, North Africa, and Turkey (MENAT) region: A tale of conflict, challenges, and opportunities

**DOI:** 10.3389/fonc.2022.1050168

**Published:** 2022-11-24

**Authors:** Zahi Abdul-Sater, Deborah Mukherji, Salim M. Adib, Ali Shamseddine, Ghassan Abu-Sitta, Ibtihal Fadhil, Richard Sullivan, Amal Al Omari, Shadi Saleh, Ali Taher

**Affiliations:** ^1^ Global Health Institute, American University of Beirut, Beirut, Lebanon; ^2^ Department of Hematology/Oncology, American University of Beirut Medical Center, American University of Beirut, Beirut, Lebanon; ^3^ Faculty of Health Sciences, American University of Beirut, Beirut, Lebanon; ^4^ Eastern Mediterranean NCD Alliance, Kuwait City, Kuwait; ^5^ Institute of Cancer Policy & Conflict & Health Research Group, King’s College London, London, United Kingdom; ^6^ King Hussein Cancer Center, Office of Scientific Affairs and Research (OSAR), Amman, Jordan

**Keywords:** cancer registration data, cancer control, population based cancer registries (PBCRs), cancer surveillance, Middle East & North Africa (MENA)

## Abstract

Cancer registration is a core component of national and regional cancer control strategies. In the Middle East, North-Africa and Turkey (MENAT) region, capacity and resources for cancer registration is variable and shaped by multiple contextual challenges. This viewpoint maps out practical recommendations around cancer registration, in an attempt to inform cancer control planning, policy, and implementation. The recommendations laid out in this viewpoint are informed by the discussions held at the Initiative for Cancer Registration in the MENAT (ICRIM) virtual workshop, which convened registry managers, policy makers, and international agencies from 19 countries in the MENAT region. The discussions were distilled in four categories of recommendations, revolving around cancer registration procedures, collaborative governance, putting cancer registration on the map, and capacity building. This viewpoint provides a much-needed mapping of practical recommendations around cancer registration, informed by direct key stakeholders in the region. These practical recommendations offer a road map for policy making, cancer control planning, and future regional capacity strengthening initiatives.

## Introduction

The global burden of cancer is disproportionately greater in low- and middle-income countries (LMICs), including many countries in the Middle East, North Africa, and Turkey (MENAT) region (**Table 1**) (1). An increase of more than 60% in cancer burden is projected in LMICs by 2030 (2). Due to multiple factors, including recurrent and endemic conflicts, changing demographics, and increased environmental contamination, the MENAT region is expected to witness the highest increase in cancer burden worldwide (3). The burden of cancer in the MENAT is multifaceted. In the Gulf Cooperation Council (GCC) countries, which include Bahrain, Kuwait, Oman, Qatar, Saudi Arabia, and the United Arab Emirates, colorectal and lung cancers are the most prevalent in men, and breast and thyroid cancers are most prevalent in women. In the rest of the MENAT region, lung, liver, and prostate cancers are the most common in men, whereas breast, colorectal, and cervical cancers are the most common in women (4). This rising burden of cancer in the region necessitates clear and well-developed national and regional cancer control plans. Accurate cancer registration is an instrumental component of any cancer control strategy (5). However, in the MENAT region, cancer registration, especially population-based, is underdeveloped, varies immensely across countries, and faces multiple logistical, political, financial, and conflict-related challenges (2, 6, 7).

**Table 1:** The socioeconomic and demographic diversity in the MENA region.

Country	Population	HDI	HDI tier	GNI per capita (US$)
Bahrain	1,472,233	0.875	Very high	39,497
Kuwait	4,268,873	0.831	Very high	52,920
Oman	4,576,298	0.816	Very high	27,054
Qatar	2,695,122	0.855	Very high	87,134
Saudi Arabia	3,640,882	0.875	Very high	46,112
Turkey	85,341,241	0.838	Very high	31,033
United Arab Emirates	9,441,129	0.911	Very high	62,574
Algeria	44,903,225	0.745	High	10,800
Egypt	110,990,103	0.731	High	11,732
Iran (Islamic Republic of)	8,855,057	0.774	High	13,001
Jordan	11,285,869	0.72	High	9,924
Lebanon	5,489,739	0.706	High	9,526
Libya	6,812,341	0.718	High	15,336
Palestine	5,250,072	0.715	High	6,583
Tunisia	12,356,117	0.731	High	10,258
Iraq	44,496,122	0.686	Medium	9,977
Morocco	37,457,971	0.683	Medium	7,303
Syrian Arab Republic	22,125,249	0.577	Medium	4,192
Mauritania	4,736,139	0.556	Medium	5,075
Djibouti	1,120,849	0.509	Low	5,025
Sudan	46,874,204	0.508	Low	3,575
Yemen	33,696,614	0.455	Low	1,314
Somalia	17,597,511	—	Not rated	1,018

HDI, Human Development Index; GNI, gross national income; MENA, Middle East and North Africa.

While countries in the MENAT region share sociocultural attributes and history, they have very diverse socioeconomic and political contexts that impact their health and the health systems in place (**Table 1**). Rich countries like Qatar, Saudi Arabia, and the United Arab Emirates invest heavily in health infrastructure and developing medical cities and complexes, which raised the quality of healthcare services (8). Countries like Algeria, Egypt, Iraq, Jordan, and Lebanon have made significant progress in improving health outcomes but are still facing multiple political, economic, and social challenges that render their health systems fragile and unable to cope with rising healthcare demands (9). Countries with medium and low Human Development Index (HDI), including Syria, Yemen, and Sudan, have the highest infant mortality rates and maternal mortality ratios in the MENA region. These countries have been plagued by recurrent and chronic conflicts, further degrading their already fragile health systems (10).

Historically, the role of cancer registration was to identify standard descriptive epidemiological parameters—incidence and prevalence—which were suggested to form the basis of understanding prevention, screening, early diagnosis strategies, and treatment: survival and mortality (11, 12). This, along with national audits, could then be utilized for wider services and systems analysis (13). Additionally, cancer registries play a significant role in the evidence-based management of cancer in some high-income countries, through the analysis of health system capacities to inform planning (14). Importantly, cancer registries can provide high-quality data for epidemiological research studies and feasibility assessment for prospective interventional clinical trials, as well as quality indicators for healthcare outcome monitoring.

## The Initiative for Cancer Registration in the MENAT virtual workshop

*The Lancet* series on global oncology emphasized the need for capacity strengthening in cancer registration across the MENAT region, for involving stakeholders in the process of cancer surveillance and for facilitating networking activities (2, 15–17). In harmony with this call and the established challenges around cancer registration, the Global Health Institute at AUB (AUB GHI), in partnership with the Naef K. Basile Cancer Institute (NKBCI) and the Research for Health in Conflict (R4HC-MENA) consortium, organized the Initiative for Cancer Registration in the MENAT (ICRIM) virtual workshop. The workshop gathered more than 30 cancer registry managers, academics, researchers, and clinicians from the MENAT region, including conflict-affected countries, to discuss challenges and propose recommendations around cancer registration.

Prior to the workshop, a survey was sent (in 2019) to 26 national and institutional cancer registry managers and administrators from 19 countries in the MENAT region (Algeria, Bahrain, Egypt, Iraq, Jordan, Kuwait, Lebanon, Libya, Morocco, Oman, Palestine, Qatar, Saudi Arabia, Sudan, Tunisia, Turkey, United Arab Emirates, and Yemen) to understand the current landscape of cancer registration across these very different health systems. The self-administered online survey, in both English and Arabic languages, was composed of 25 questions related to cancer registration status in the respective country (10 questions) and cancer registry-specific questions (15 questions) (**Supplementary Material**). The results highlighted the divide in cancer registration resources and capacities in the MENAT. While GCC countries reported well-developed population-based cancer registries, countries like Syria, Yemen, Libya, and Iraq reported severely hindered cancer registration due to chronic and recurrent conflicts and displaced populations. The challenges around incomplete medical records, inaccurate death records, lack of trained staff, absence of legislation mandating cancer registration, lack of funds, weak healthcare infrastructure, and poor communication between stakeholders were also reported (6). The ICRIM Workshop took place remotely on the 3 and 4 February 2022, where the participants engaged in two rounds of discussions, framed around the results of the survey, to formulate contextualized recommendations for improving cancer registration in the MENAT.

## Recommendations for improving cancer registration in the MENAT

The ICRIM discussions around improving cancer registration in the MENAT region can be distilled into the following recommendations:

1. Cancer registration procedures: To reduce the variability in registries and under-reporting and ensure the high quality of vital statistics and mortality data, cancer registration procedures and related data systems should be standardized, linked, and digitalized.
a) Establish standard operating procedures (SOPs) for cancer registries in the region, which include standardized registration forms, in addition to training, operation, ethics, and confidentiality manuals and guidelines conforming with international standards (International Agency for Research on Cancer (IARC)).
“Filing system at data sources need to be improved”—*Libya*
“Different systems for cancer data entry in the West Bank and Gaza”—*Palestine*
“Committee to standardize cancer registration methodology for the region; “Development of a standardized report form for cancer registration”—*Egypt*
“Design a Standard Procedure Manual for cancer registries”—*Oman*
b) Connect cancer registration records with civil and death registries to ensure proper data linkage.
“Link cancer registry with civil registration for demographic data”—*Palestine*
“Linkages of PBCR with other data sets, and promote data use in cancer control plans (patient satisfaction, medical expenditures, quality of life)”—*Turkey IARC Hub*
2 Collaborative governance: regional networks must be mobilized to establish a collaborative governance structure that includes key stakeholders in the region. This was a cross-cutting theme highlighted by most attendees.
a) Create a common legal framework for reporting and registration. In our survey, 8 out of 22 registries (from 19 countries) reported an absence of legislation mandating cancer registration in their country.
“Legislations mandating cancer registration: Cancer is not a notifiable disease”—*Libya*
“Create a legal framework for reporting and registration”—*Yemen*
“Decree that mandates cancer registration for each country”—*Egypt*
“Decree to notify cancer cases by all stakeholders, aiming to achieve a population-based cancer registry and quality network at national level”—*Syria*
b) Establish formal governmental linkages with cancer registration agencies under the World Health Organization (WHO), including International Association of Cancer Registries (IACR) and Global Initiative for Cancer Registry Development (GICR). Alternatively, IARC may reach out directly to national cancer registry departments to support logistical improvements while avoiding arduous red tape in heavy bureaucratic pathways.
“Lack of Major international initiatives aiming at developing or improving cancer registration (e.g., ARC/GICR site visits, training workshops, cancer registration initiatives, etc.)”—*Tunisia*
“Collaborate with IARC/GICR (WHO) to conduct more training for cancer registrars on data quality and cancer staging”—*Oman*
*“*Establishment of a professional collaboration with WHO, IARC on cancer registration and research”—*United Arab Emirates*
c) Introduce registry twinning programs, linking MENAT registries with established regional and global registries. This would facilitate the effective exchange of expertise, skills, and knowledge.
“More interaction between the registries in different countries, meetings for cancer registrars, site visits; Registry twinning programs”—*Turkey*
“Networking and twining”—*Jordan*
d) Establish a regional cancer registration expert group for sharing expertise and knowledge. The registry expert group would form a cancer registry scientific coordination committee that aims to ensure the proper governance, implementation, and financial sustainability of cancer registration in the region.
“Establish a Cancer Registry Scientific Coordination Committee”—*Yemen*
*“*Set up a MENA Scientific Committee”—*Algeria*
“Regional networking, and advocacy to increase cancer awareness and political commitments to cancer surveillance”—*Morocco*
3. Putting cancer registration on the map: a bold approach must be adopted to increase visibility, awareness, and productive collaboration on cancer registration in the MENAT and its impact on practice and policy.
a) Establish an online knowledge exchange and convening platform to bring together relevant multi-sectoral stakeholders in cancer registration to develop a “community of practice”. The platform would organize regular regional webinars and workshops that approach, in a contextualized manner, topics including staff retention strategies, technical aspects, economic evaluation, and awareness around cancer registration. This platform may help maximize the dissemination and use of data and knowledge by engaging different stakeholders, including communities, and using various communication channels that can be tailored and contextualized to different audiences.
“Lack of trained staff and difficulty in trained staff retention”—*Turkey IARC Hub*
“Scientific collaboration: studies, publications”—*Turkey*
“Meeting for the key officials to explain importance of cancer registration”—*Egypt*
“Advance workshops and hands on training in specific areas on cancer registration, such as data analysis and scientific writing, quality control, cancer staging”—*United Arab Emirates*
“Regional workshops to share experience and lessons learnt”—*Lebanon*
b) Institute an annual “Day of Cancer Registration” in the MENAT. This day would be attached to an event, where cancer registrars, epidemiologists, policymakers, researchers, and clinicians convene to discuss cancer registration needs, challenges, and recommendations. Such events would include technical, policy, and dissemination meetings to create a translation pipeline from knowledge to policy.
“Plan an annual International Day of MENA cancer registries”—*Algeria*
“Look for pathways to influence decision-makers”—*Turkey*
c) Develop social media campaigns on cancer registration in Arabic, English, French, and Turkish languages. In addition, documentation should be produced to raise awareness on cancer registration targeting the general population and policymakers in the MENAT region.
“Increase awareness advocacy and marketing of cancer registration”—*Jordan*
“Raising awareness to the indispensability of accurate cancer surveillance for cancer control”—*Morocco*
4. Capacity building: capacity building for cancer registration should be expanded to train more data entry staff to utilize electronic systems and ensure proper data capture (using CanReg5 at minimum). This theme was particularly pertinent to LMICs in the MENAT region, including Palestine and Iraq. Cancer registration capacity was noted to
a) Implement regular capacity-strengthening trainings by the GICR to all registries in the region. Such training should be expanded to include all registry staff, in addition to training of other key stakeholders including staff at the ministries of health and at sources of data (i.e., centers where cancer is diagnosed and treated).
“Training of focal points on CanReg5 and abstracting data”—*Palestine*
“Continuous visits and evaluation by International Agency for Research on Cancer (IARC)/(GICR)”—*Iraq*
“Expand cancer registration capacity of the existing centers through the training of personnel and upgrading of facilities”—*Yemen*
“We need workshops at all levels, at Ministry level and at medical level”—*Yemen*
“Provide regular training on cancer registration”—*Algeria*
b) Organize an easily accessible and contextualized certification program for cancer registrars. Advanced training for key staff would be undertaken to establish peer to peer “trainers” team within the region.
“None of the cancer registry personnel is a Certified Tumor Registrar”—*Palestine*
“Provide advanced training for the key staff to establish a ‘trainers team’”—*Iraq*
“Training workshops to train registers and improve the quality of reporting”—*Sudan*
c) Develop an online course for cancer registration, taking into account local and regional challenges. The course would be asynchronous and would provide the basis for developing a cancer registration certificate.
“Provide basic Cancer Registration Training Programs for all staff. A structured certification program might be organized”—*Iraq*

In conclusion, the workshop revealed a profound need among those involved in cancer registration in MENAT for increased visibility, training opportunities, and political support from international entities and for developing regional collaboration and cooperation. In the discussions, it was clear that the needs and challenges were most severe in countries impacted by conflict. Further convening events, including workshops, meetings, and conferences, are needed to garner the necessary support and buy-in from cancer registration stakeholders to materialize the recommendations that were laid out in this article. ICRIM has been mandated to continue its leading role on these issues in collaboration with all stakeholders in the region.

## ICRIM

Wafaa Al Tajjar, World Health Organization; Ali Saeed Alzahrani, Gulf Centre for Cancer Control and Prevention, Saudi Arabia; Khadija Abu Khade, Palestinian National Institute of Public Health, Occupied Palestinian Territories (OPT); Karima Bendahou, Ministry of Health, Morocco; Mokhtar Hamdi Cherif, Setif Cancer Registry, Algiers; Khitam Mohsen, Ministry of Health, Iraq; Razan Yassin, Sudan Cancer Registry, Sudan; Amany Elbasmy, Kuwait Cancer Registry, Kuwait; Feras Al Jerf, Syria National Cancer Registry, Syria; Heba Fouad, World Health Organization; Huda Lahham, Palestinian Ministry of Health, Occupied Palestinian Territories (OPT); Huda Basaleem, Aden Cancer Registry; Kaveh Khoshnood, Yale University, USA; Khaled Jamal, King Hussein Cancer Center (KHCC), Jordan; Mahmoud Daher, World Health Organization; Mona Numairy, National Cancer Registry, Sudan; Manal Mohamed Ali, Bahrain cancer registry, Bahrain; Mohamed Hsairi, National Registry of Tunisia, Tunisia; Nabiel Nazmi, National Cancer Registry of Egypt; Omar Nimri, Ministry of Health, Jordan; Suad elnaami, National cancer registry, Libya; Sultan Eser, Izmir Cancer Registry, Turkey; Tezer Kutluk, Pediatric Cancer Registry, Turkey; Eman Hasan Janahi, Ministry of Health, Bahrain; Waled Masaud, National Cancer Control Program, Libya; Wael Shelpai, Ministry of Health, United Arab Emirates; Najla allawati, Ministry of Health, Oman.

## Data availability statement

The original contributions presented in the study are included in the article/**Supplementary Material**. Further inquiries can be directed to the corresponding author.

## Author contributions

ZA-S: conceptualizing, writing-original draft, supervision, writing-review and editing, project administration. DM: conceptualizing, writing-review and editing, supervision; SA: conceptualizing, writing-review and editing, supervision; AS: conceptualizing, writing-review and editing, supervision; GA-S: conceptualizing, writing-review and editing, supervision; IF: conceptualizing, writing-review and editing, supervision; RS: conceptualizing, writing-review and editing, supervision, funding acquisition; AA: conceptualizing, writing-review and editing, supervision; SS: conceptualizing, writing-review and editing, supervision, funding acquisition; AT: conceptualizing, writing-review and editing, supervision, funding acquisition. All authors contributed to the article and approved the submitted version.

## Funding

This work was supported by UK Research and Innovation as part of the Global Challenges Research Fund and Research for Health in Conflict in the Middle East and North Africa (R4HC-MENA) project, grant number ES/P010962/1. The sponsor had no involvement in the development or conceptualizing of the work.

## Conflict of interest

The authors declare that the research was conducted in the absence of any commercial or financial relationships that could be construed as a potential conflict of interest.

## Publisher’s note

All claims expressed in this article are solely those of the authors and do not necessarily represent those of their affiliated organizations, or those of the publisher, the editors and the reviewers. Any product that may be evaluated in this article, or claim that may be made by its manufacturer, is not guaranteed or endorsed by the publisher.
