# Supplementary material for: Cancer registration in the Middle East, North Africa, and Turkey (MENAT) region: A tale of conflict, challenges, and opportunities
Source: Front Oncol. 2022 Nov 24;12:1050168. doi: 10.3389/fonc.2022.1050168 (PMC9730320; doi:10.3389/fonc.2022.1050168)
Supplement: Supplementary file 1 [file DataSheet_1.pdf]

This survey has been published in the following article: Abdul-Sater, Zahi, et al. "Cancer Registration in the Middle East, North Africa, and Turkey: Scope and Challenges." JCO Glob. Oncol., vol. 7, July 2021, pp. 1101-09, doi:10.1200/GO.21.00065.

Thank you for taking the time to complete this survey, which aims to inform the ICRIM Workshop. The ICRIM workshop is convened by the Global Health Institute at AUB, in collaboration with Naef K. Basile Cancer Institute (NKBCI), Children's Cancer Center of Lebanon (CCCL), and Research for Health in Conflict in the MENA (R4HC-MENA). It aims to systematically assess the landscape of cancer registration and discuss strategies to improve cancer registration in the MENAT region.

The survey is divided into two sections:

- Section 1 comprises of general questions about cancer registration in the country.
- Section 2 comprises of registry-specific questions (institutional and/or subnational).

**Please press next if you voluntarily agree to be a part of this research. This will take you to the survey questions. If you do not wish to be a part of this research, please close this page.**

You can view the full text of the consent form **here (https://docs.google.com/document/d/1e5_EBVcu4cd5Dn3roQS31sXCguvmQFIVl3XLbq2NWJ0/edit?usp=sharing)**.

There are 23 questions in this survey.

# Cancer registration in your country

Please answer the following questions that pertain to the status of cancer registration in your country.

## Country: *

❗ Choose one of the following answers

Please choose **only one** of the following:

- ◯ Algeria
- ◯ Bahrain
- ◯ Egypt
- ◯ Iraq
- ◯ Jordan
- ◯ Kuwait
- ◯ Lebanon
- ◯ Libya
- ◯ Morocco
- ◯ Oman
- ◯ Palestine
- ◯ Qatar
- ◯ Saudi Arabia
- ◯ Sudan
- ◯ Syria
- ◯ Tunisia
- ◯ Turkey
- ◯ United Arab Emirates
- ◯ Yemen
- ◯ Other ____________

## Please select the most relevant state and data collection method of the following types of cancer registries in your country.

Please choose the appropriate response for each item:

| | Not developed | Partially developed | Well developed | Not sure |
|---|---|---|---|---|
| **Pathology-based cancer registries** | ○ | ○ | ○ | ○ |
| **Hospital-based cancer registries** | ○ | ○ | ○ | ○ |
| **Population-based cancer registries** | ○ | ○ | ○ | ○ |

## Please specify the number of the below registries in your country.

❗ Only numbers may be entered in these fields.

| | Number of Registries |
|---|---|
| **Pathology-based registries** | |
| **Hospital-based registries** | |
| **Population-based registries** | |
| **Registries collecting cancer survival data** | |

## Is reporting cancer cases to the cancer registry mandated by law in your country? *

Please choose **only one** of the following:

- ◯ Yes
- ◯ No

## Please describe key challenges of cancer registration in your country *(eg. Funding, Trained staff, National Policies, etc.)*. Kindly list them in order of importance.

| | Challenge Description |
|---|---|
| **Challenge 1** | |
| **Challenge 2** | |
| **Challenge 3** | |
| **Challenge 4** | |
| **Challenge 5** | |

## Did any conflict-related events (Refugee influx, armed conflict, etc.) affect the process of cancer registration in your country? *

Please choose **only one** of the following:

- ◯ Yes
- ◯ No

## Please date those events and detail their effects on the cancer registration process in your country.

Only answer this question if the following conditions are met:
Answer was 'Yes' at question ' [Q00007]' (Did any conflict-related events (Refugee influx, armed conflict, etc.) affect the process of cancer registration in your country?)

| | **Date of Event** | **Description of Event and its effect on cancer registration** |
|---|---|---|
| **Conflict Event 1** | | |
| **Conflict Event 2** | | |
| **Conflict Event 3** | | |
| **Conflict Event 4** | | |
| **Conflict Event 5** | | |
| **Conflict Event 6** | | |

# Registry-specific status of cancer registration

Please answer the following questions that pertain to the status of the registry that you manage.

## Registry (official name) *

Please write your answer here:

## Year of registry establishment

Please enter a date:

## Type of Registry *

Choose one of the following answers

Please choose **only one** of the following:

- Population-based
- Hospital-based
- Pathology-based
- Other

## Data Collection Method *

Choose one of the following answers

Please choose **only one** of the following:

- Active *(registry staff regularly visits hospitals or other institutions)*
- Passive *(Notification of diagnosed cases is sent to the registry on a routine, continuing basis)*
- Mixed *(Active and Passive)*
- Automated
- Other

## Approximately, what is the frequency of data collection *(In days)*?

Only numbers may be entered in this field.

Please write your answer here:

## Please indicate which of the available data sources at the registry are in electronic form and which are paper-based. If the record type is not used, please select "not used".

Please choose the appropriate response for each item:

| | Electronic | Paper | Not used |
|---|---|---|---|
| **Hospital discharge records** | ◯ | ◯ | ◯ |
| **Histopathology records** | ◯ | ◯ | ◯ |
| **Cytopathology records** | ◯ | ◯ | ◯ |
| **Hematology records** | ◯ | ◯ | ◯ |
| **Radiation oncology records** | ◯ | ◯ | ◯ |
| **Medical oncology records** | ◯ | ◯ | ◯ |
| **Death records** | ◯ | ◯ | ◯ |
| **Autopsy records** | ◯ | ◯ | ◯ |
| **Hospital medical records** | ◯ | ◯ | ◯ |
| **Hospice records** | ◯ | ◯ | ◯ |
| **Private hospital records** | ◯ | ◯ | ◯ |
| **Radiology records** | ◯ | ◯ | ◯ |
| **Primary care records** | ◯ | ◯ | ◯ |
| **Other cancer registries** | ◯ | ◯ | ◯ |

## Does the registry have procedures are in place to minimize the risk of duplicate registration?

Please choose **only one** of the following:

- Yes
- No

## Please select the number of full-time staff that currently work at the cancer registry?

Choose one of the following answers

Please choose **only one** of the following:

- 0-5
- 5-10
- 10-15
- >15
- Don't know

## Does the registry arrange for training new staff and continuous professional of existing staff?

Please choose **only one** of the following:

- Yes
- No

## Please select the data type collected by the registry on the **country population.**

❗ Check all that apply

Please choose **all** that apply:

- ☐ Administrative unit *(Geographical area)*
- ☐ Population size
- ☐ Distribution of coverage by Age
- ☐ Distribution of coverage by Socio-economic status
- ☐ Distribution of coverage by Urban-rural residence

## Does the registry collect information on non-nationals *(eg. Refugees or Foreign Nationals)*

Please choose **only one** of the following:

- ◯ Yes
- ◯ No

## Please describe the non-national population that the registry collects information on.

Only answer this question if the following conditions are met:
Answer was 'Yes' at question ' [Q00019]' (Does the registry collect information on non-nationals (eg. Refugees or Foreign Nationals))

Please write your answer here:

## Which application software is used for registration at the registry?

Please write your answer here:

## Please describe any major changes in the operation of the registry since its establishment *(eg. death records becoming routinely available)*. Kindly provide the date and a decription of the corresponding event.

| | Date of Event | Description of Event |
|---|---|---|
| **Major Event 1** | | |
| **Major Event 2** | | |
| **Major Event 3** | | |
| **Major Event 4** | | |
| **Major Event 5** | | |
| **Major Event 6** | | |

## Please attach any document (s) that you think is (are) relevant to this questionnaire. These may include organizational charts, coverage maps, data flow diagram, etc...

Please upload at most 10 files

Kindly attach the aforementioned documents along with the survey

## Please feel free to add any comment or additional information about your registry that you think is relevant.

Please write your answer here:

Disclaimer:
Registry-specific Questions in this survey were adopted from: Standards and Guidelines for Cancer Registration in Europe. IARC Technical Publication No. 40. Appendix 2: Structured reviews of cancer registries. Authors: Tyczynski JE, Démaret E, Parkin DM
Country-wide registration questions were adopted from "A survey of participants and experts" conducted at "Regional Meeting on Cancer Control and Research Priorities in the Eastern Mediterranean Region". A. Shamseddine

Thank you for filling out the survey. The information you provided will help towards a successful cancer registration workshop.

22.08.2022 – 02:24

Submit your survey.
Thank you for completing this survey.
